# Influenza Vaccine Uptake and Associated Hospitalization Risk in Older Adults with or Without Dementia: Differences Between at Home-Living and Nursing Home Residents in Lombardy, Italy

**DOI:** 10.3390/vaccines13050489

**Published:** 2025-04-30

**Authors:** Lorenzo Blandi, Carlo Signorelli

**Affiliations:** School of Public Health, Vita-Salute San Raffaele University, Via Olgettina 58, 20132 Milan, Italy; signorelli.carlo@hsr.it

**Keywords:** influenza, respiratory diseases, dementia, older people, nursing homes, vaccination, vaccine hesitancy

## Abstract

Objective: Our population-based cohort study aims to compute the uptake of the influenza vaccine and the associated risk of hospitalization for respiratory diseases of infectious origin based on the residency setting and dementia status of people aged 65 or over. Methods: We conducted a retrospective cohort study on the whole population of residents aged ≥65 in Lombardy, the most populated Italian region. Using region-wide administrative data, we computed the seasonal prevalence of vaccination for influenza from 1 October 2022 to 30 April 2023. To estimate the risk of hospitalization, we applied Fine-Gray sub-distribution hazard models, accounting for the competing risk of death and adjusting for confounders. Results: Our study analyzed 2,420,279 individuals aged 65+ in Lombardy. Overall, 51.4% received an influenza vaccination in 2022–2023. Among residents living at home, 50.8% were vaccinated, while nursing home residents had an uptake of 74.0%. People living with dementia reported a vaccination coverage of 62.6%, and vaccination rates were higher among those residing in nursing homes than those who lived at home. The adjusted sub-hazard ratios (SHRs) showed higher hospitalization risks of 1.88 for unvaccinated individuals with dementia and 1.74 for unvaccinated individuals without dementia living at home. In nursing homes, the SHR for respiratory hospitalization was 2.20 for individuals without dementia and 2.40 for dementia patients. Vaccination reduced risks across all groups, but disparities persisted. Conclusions: People living with dementia were more likely to be hospitalized for respiratory diseases. However, they reported an influenza vaccination coverage that was below expectations and similar to the general population, both in nursing homes and home-living settings. Public health institutions should extend and mention dementia as a higher-risk condition.

## 1. Introduction

Influenza is a major global health concern, particularly for older adults and individuals with chronic illnesses, who are at an increased risk of severe complications [1,2]. In fact, most influenza-related fatalities occur in people aged 65 and older worldwide [3]. The elderly are more likely to be admitted to hospitals, face respiratory or non-respiratory complications related to influenza, or even die [3]. The World Health Organization has set the influenza vaccination coverage target for people over 65 at 75% [4]. Vaccination is widely recognized as an effective measure to reduce illness severity and prevent hospitalizations in this vulnerable population [3,5]. However, dementia and cognitive impairments such as memory loss and limited health awareness may reduce the likelihood of vaccination within this group. Hence, the association between dementia and influenza vaccine uptake has been widely studied, but findings have been inconsistent. Some research suggested that individuals with dementia or cognitive impairment may have higher vaccination rates compared to those without [6,7], whereas other studies indicate no significant difference or even lower uptake [8,9]. Furthermore, recent evidence suggested that the effect of dementia on vaccination rates may differ between individuals residing at home and those residing in nursing homes [10,11]. These findings were retrieved in Denmark and the United Kingdom, where unexpectedly elevated vaccination rates among dementia patients in nursing homes and reduced community uptake were found. These authors reported that reduced community uptake was more likely attributable to organizational reasons in their countries rather than patient or clinical factors, and identifying and comprehending the obstacles to uptake in each health system is crucial for enhancing care for dementia patients and increasing general community vaccine uptake [10,11]. All these discrepancies might derive from methodological limitations such as small sample sizes, disease-specific study populations, or reliance on self-reported data. A recent scientific project known as “EPIDEM” was approved by the regional health institution and is based on the administrative data of a population-based and region-wide Italian dataset. It succeeded in identifying populations living with dementia through a validated algorithm [12], estimating their incidence, mortality, and hospitalization trends over the last 20 years [13,14].

Given these gaps in the literature, there is a need for large-scale, representative studies. By using the same dataset and methodological approach as EPIDEM and considering residency in nursing homes and other critical variables, it is possible to provide a more comprehensive understanding of influenza vaccination patterns among older adults with and without dementia, as well as their impact on hospitalization due to respiratory diseases. Our hypothesis is that influenza vaccination coverage is still too low and that further preventive interventions are needed to mitigate the burden of respiratory diseases, especially among people living with dementia. Indeed, respiratory diseases are the main causes of hospitalization among people living with dementia [14,15], despite Italy’s prevention efforts, such as setting the same target of influenza vaccination coverage as the WHO for the elderly [16]. Thus, our population-based study aims to compute influenza vaccine uptake and the associated risk of hospitalization for respiratory diseases of infectious origin based on the residency setting and dementia status of people aged 65 or over.

## 2. Methods

### 2.1. Study Design

We conducted a retrospective cohort study on the whole population of residents aged ≥65 in Lombardy (2,420,279 people), the most populated Italian region, using regional administrative data from multiple sources and covering the 2022–2023 influenza season.

### 2.2. Data Sources and Setting

Administrative data are collected by the Lombardy Region Welfare Directorate in its comprehensive healthcare system’s Data Warehouse (DWH). DWH has a star scheme model, characterized by a central fact table (the Population Registry dataset) surrounded by a set of dimensional tables (all administrative informational flows), thus enabling detailed longitudinal analyses at the individual level. The Population Registry includes sociodemographic information and mortality data. The administrative information flows contain records from various sources, including the hospital discharge registry, nursing home admissions, drug prescriptions, outpatient services, and other healthcare records documented by the regional healthcare system. This ensures that all citizens’ interactions with the healthcare system are comprehensively captured, allowing for a full historical view of their health status and medical treatments. The present study is part of the “EPIDEM” project, approved by the Lombardy Welfare Directorate in 2024. Following the privacy-by-design principles, this project was approved by a regional committee, which allowed access to a selection of region-wide administrative and health data. All data handling procedures complied with the European General Data Protection Regulation (GDPR) and local privacy laws to protect the confidentiality and integrity of citizens’ information.

### 2.3. Study Population and Follow-Up

Our study included residents of Lombardy aged 65 or over at the study’s baseline (born in 1957 or before). The study period covered the seasonal influenza campaign from 1 October 2022 to 30 April 2023. To identify people living with dementia, we used a validated algorithm based on administrative data, published by the Italian National Health Institute [12]. A case of dementia was defined as having at least two different prescriptions for dementia drugs within 12 months OR at least one hospital discharge with primary or secondary diagnoses of dementia OR if the subjects had an exemption from healthcare co-payments specifically for the disease OR if the resident was in an LTCF with reported cognitive deficits [12,13]. Thus, we selected the following four different information flows (see Supplementary Materials, Table S1):

(i) Hospital discharge registry;
(ii) Drug prescriptions;
(iii) Administrative exemptions (administrative certificates for selected medical conditions that are exempt from payment for health services);
(iv) Long-term care facility admissions.

The proportion of people living with dementia (PLWD) identified from different data sources is presented in the Supplementary Materials (see Figure S1).

To identify people living in nursing homes, we considered only those admitted before or during the study period, with at least a 105-day length of stay (half of the follow-up period), retrieving them from the long-term care administrative data.

### 2.4. Variables of Interest

The exposure of interest was the vaccination status for influenza. We retrieved this information from the vaccination information flow, including those vaccinated over the study period.

The outcome of interest was the rate of hospitalization with respiratory diseases of infectious origin. Thus, we included only pneumonia and bronchitis diagnoses attributable to an infectious origin, according to the ICD9-CM classification (480–491 diagnosis codes as primary or secondary causes at the time of discharge, i.e., 480.0, 480.1, 480.2, etc.), as influenza can occur as a primary infection or a concurrent infection. We implemented left truncation, excluding those individuals in whom the outcome occurred prior to the exposure.

As covariates, we included a history at baseline of the main condition and diseases identified as risk factors for respiratory diseases: chronic obstructive pulmonary disease and diabetes (both retrieved according to the official Lombardy algorithms for DWH [17] from the following information flows: drug prescriptions, the hospital discharge registry, and administrative exemptions) and immunocompromised status (according to the algorithm of the Agency for Healthcare Research and Quality).

### 2.5. Statistical Analysis

We computed the seasonal prevalence of people vaccinated for influenza, both overall and stratified by sex, residence setting (living at home or nursing home residents), and dementia status, from 1 October 2022 to 30 April 2023.

To estimate the risk of hospitalization with respiratory diseases in people who were not vaccinated, we applied Fine-Gray sub-distribution hazard models, accounting for the high competing risk of death in an older and clinically vulnerable population. This model provides more accurate and interpretable estimates of the association between influenza vaccination status and hospitalization risk for respiratory diseases. These models were stratified by sex and age and adjusted for selected potential confounders, including age, a history of chronic obstructive pulmonary disease, diabetes, and immunocompromised status. The results were reported as sub-distribution hazard ratios (SHRs) with 95% confidence intervals (CIs). Fine-Gray models were performed separately to compare individuals not vaccinated vs. vaccinated individuals among people living with and without dementia, both those living at home and nursing home residents. All the analyses were performed using the statistical software SAS 9.4.

## 3. Results

Our study included 2,420,279 people aged 65 or over and living in Lombardy (see Table 1). A total of 51.4% were vaccinated for influenza during the 2022–2023 season. A total of 2,352,023 resided at home and 50.8% were vaccinated. A total of 68,256 individuals lived in nursing homes, with a vaccination coverage of 74.0%.

Within our population, we identified 94,268 people living with dementia. About 62.6% of them received an influenza vaccination. Stratifying by residency setting and sex (see Table 2), a total of 17,841 females (51.4%) and 10,778 males (56.1%) living at home were vaccinated. On the other hand, we found a higher vaccine uptake in nursing home residents, where 24,071 (76.3%) females and 6327 (72.1%) males received the vaccination.

Regarding people without dementia, we identified 2,326,011 individuals. Approximately 51.0% received the immunization. Stratifying by residency setting and sex (see Table 3), a total of 638,075 (50.4%) females and 527,591 (51.1%) males living at home were vaccinated. Among nursing home residents, the uptake was higher, with 14,782 (72.9%) females and 5360 (70.2%) males vaccinated.

**Figure 1:**
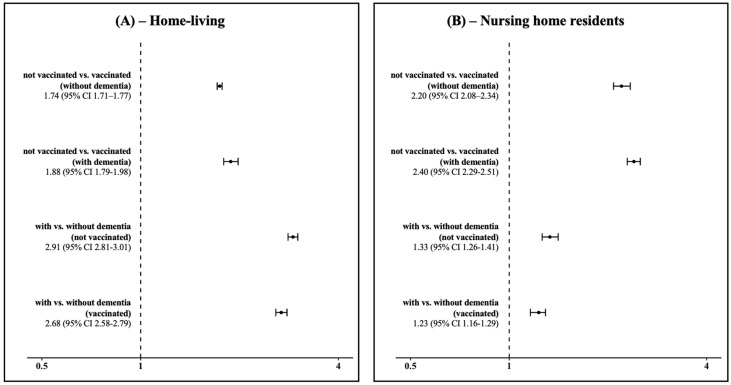
Sub-distribution hazard ratios (SHRs) of hospitalization with respiratory diseases and corresponding 95% confidence intervals in people living at home (panel (**A**)) and in nursing home residents (panel (**B**)) in Lombardy, Italy, over the 2022–2023 influenza season.

Subsequently, we computed the adjusted SHRs of hospitalization with respiratory diseases. Comparing people who were not vaccinated vs. vaccinated and living at home (see Figure 1, panel A), the adjusted SHR was 1.74 (95% CI 1.71–1.77) in people without dementia, and 1.88 (95% CI 1.79–1.98) in people living with dementia. The adjusted SHR for adults living at home with dementia compared to those without dementia was 2.91 (95% CI 2.81–3.01) for the unvaccinated group and 2.68 (95% CI 2.58–2.79) for the vaccinated group.

Comparing people not vaccinated vs. vaccinated residing in nursing homes (see Figure 1, panel B), the adjusted SHR was 2.20 (95% CI 2.08–2.34) in people without dementia, and 2.40 (95% CI 2.29–2.51) in people living with dementia. When comparing people without dementia vs. those with dementia residing in nursing homes, the adjusted SHR was 1.33 (95% CI 1.26–1.41) in individuals who were not vaccinated, and 1.23 (95% CI 1.16–1.29) in vaccinated individuals.

## 4. Discussion

Our research was performed during the influenza season of 2022–2023, using administrative data. We quantified the impact of influenza on raising the risk of hospitalization with respiratory disorders by conducting a population-based study on adults aged 65 or older who resided in Lombardy. This study took into account the vaccination status of each individual, as well as the diagnosis of dementia. We also investigated the vaccination rates of people living with and without dementia, including those who resided in nursing homes and those who lived at home.

From our findings, it emerged that people living with dementia consistently showed a higher risk of hospitalization with respiratory diseases than those without dementia, regardless of their vaccination status. Several studies from other countries found that older adults with dementia were at an increased risk of serious infections and related morbidity and mortality compared to those without dementia [18,19]. Recent studies from Italian population-based cohorts reported that respiratory diseases were the main cause of hospitalizations since 2017 [14], and an increase in mortality trends occurred among people living with dementia over the 2013–2023 period [13]. Our results were consistent with a higher risk of hospitalization among people living with dementia. Indeed, among individuals living at home, the risk increased by 168% for those vaccinated and 191% for those not vaccinated. Among nursing home residents, the risk was lower but still exceeded 23% for those vaccinated and 33% for those not vaccinated, perhaps because the nursing homes in Lombardy have medical doctors within the facility, and so they only hospitalize the most serious cases. In addition, the risk for those not vaccinated vs. those vaccinated in nursing homes was higher by 140%. Our findings suggest that dementia is an important and impactful risk factor for respiratory diseases, including influenza. We already know that people living with dementia are especially susceptible to severe infections and their associated complications [14,18,19]. However, despite the potential benefits of influenza immunization for this group, many national vaccination programs do not specifically prioritize dementia as a high-risk condition [10].

Despite the increased hospitalization risk associated with dementia, even among vaccinated individuals, our findings reported influenza vaccination coverage rates that were below expectations and highlighted the need for tailored preventive strategies. The World Health Organization has set the influenza vaccination coverage target for people over 65 at 75%, but few countries worldwide and in Europe have reached this goal [4,20,21]. Indeed, vaccination uptake in Europe ranged from 0.03% to 76.3%, with a median of 34.4%, and it was achieved only by the Netherlands and Scotland [22]. Therefore, many countries in Europe have strengthened their influenza vaccination programs [23], including Italy, where influenza vaccination is free of charge and recommended in older people [24]. Italy has set a minimum target of 75% and an optimal target of 95%, but it has never achieved these goals over the last 20 years, and the influenza vaccine coverage decreased after the pandemic [16]. Our study found that Lombardy, despite reporting a historical coverage similar to Italy [16], never reached the minimum goal. Indeed, only 51.4% of the older population were vaccinated, while people living with dementia exhibited a coverage of 62.6%. From a public health perspective, it is essential to strengthen influenza vaccination campaigns with tailored interventions for dementia. These could include targeted vaccination campaigns for individuals with dementia, ensuring optimal uptake and timely administration while also innovating service delivery [25]. Indeed, bringing people closer to the health system, for example, by involving pharmacies as vaccination centers, contributed greatly to increasing the influenza vaccination coverage [26]. Other successful experiences in different healthcare settings were reported, including vaccination during hospital stays or at discharge [27], as people with dementia are at greater risk of hospitalization. In addition, education and support programs for caregivers and health workers are needed to counteract vaccine hesitancy [28], and they can encourage primary and secondary preventive strategies against respiratory illnesses in individuals with dementia. According to the Vaccine Confidence Project [29], throughout the EU, general vaccine trust has diminished since 2020, remaining somewhat equivalent to levels observed in 2018. From 2018 to 2022, significant polarization emerged between older and younger demographics regarding vaccine perceptions, with individuals over 65 exhibiting increased confidence while those aged 18–34 displayed diminished confidence. The seasonal influenza vaccine is the sole vaccination exhibiting an inverse trend, characterized by a diminishing gap between older and younger age groups, with heterogeneous acceptance between countries due to a lack of confidence in the safety, effectiveness, and importance of the vaccine.

Nursing home residents were the most vaccinated (vaccination coverage of 74.0%), but this group did not reach the minimum goal for influenza vaccination coverage. Another Danish study recently reported data about the entire Danish elderly population, showing that nursing home residents both with and without dementia were more likely to be vaccinated than those living at home [10]. This was a repeated cross-sectional study based on administrative data from 2002/03 to 2018/19. The authors considered the vaccination status from September to August of each season, performing a multivariate logistic regression analysis. In addition, a French study based on a small population of 6275 nursing home residents aged 65 or over found higher flu vaccination coverage among people with dementia [6]. This was a cross-sectional study within the 2010/11 season. By contrast, our results in nursing homes, when we stratified by dementia status, reported similar vaccine uptake among individuals with and without dementia. We considered the vaccination only in the time interval of October to April, as this is when the Italian preventive campaign against the flu is ongoing. We performed a Fine-Gray model to take into account the high competing risk of death in an old and vulnerable population. Our findings were consistent with a UK cross-sectional study on a population of 387,568 over the 2008/09 season [11]. The higher uptake in a nursing home setting could be explained by vaccination programs and healthcare monitoring for nursing home residents, especially in Lombardy, but not for people living with dementia [30]. Nursing homes assist their patients with their specific needs on a daily basis and can protect them from the risk of hospitalization. The government and public health institutions should consider allocating specific resources to improve preventive interventions for nursing home residents with and without dementia.

Our findings highlighted that Italian public health institutions should consider people living with dementia as a target group of primary importance. Indeed, they have an increased risk of hospitalization for respiratory diseases, even if they have a similar influenza vaccination coverage to the rest of the population, both for those living at home and for those residing in nursing homes. Studies from other European countries found a higher flu vaccination coverage among people living with dementia [6,10], while a UK study reported similar findings to ours [11]. In Italy, the last Ministry of Health’s regulations regarding influenza vaccination campaigns did not mention people living with dementia as a specific group to target [20]. Our results support the fact that such people need to be paid more attention to in vaccination policies. Indeed, Italians show a high degree of vaccine acceptance towards the seasonal influenza vaccination, with more than 80% of the population stating that it is important, safe, and effective [29]. Fostering public health strategies could prevent an increase in hospitalizations, an increase in the workload on emergency departments, and a reduction in the burden of illness, especially during the flu season.

Our study has several strengths. To the best of our knowledge, our study provides the largest cohort in the literature and the first population-based data in Italy about influenza vaccination coverage in the elderly, stratifying by residency setting. We included the whole population of those aged 65 or over and residing in Lombardy and explored one of the largest cohorts in the literature on this topic. Other similar studies considered smaller sample sizes, from about 5000 to 500,000 [6,10,11]. Our study reported the first Italian data in the literature about flu vaccination coverage in nursing home residents. This is pivotal, as during COVID-19, these structures were unprepared and hosted the most vulnerable population, and we have no information about the implementation and outcomes of preventive interventions in these facilities after the pandemic. In addition, our study was the first to evaluate the population living with dementia, highlighting under-investigated and preventable disparities. Our model considered the high competing risk of death in this old and fragile population by using a Fine-Gray model. We adjusted the model for specific covariates that are implicated in an increased risk of hospitalization with respiratory diseases. We checked for differences between the unadjusted and adjusted models. The data source allowed us to retrieve administrative data on all health services provided for the entire population covered by the Italian National Health Service.

Our article also has some limitations. Our study is retrospective and based on administrative data used for secondary purposes. This could lead to underdiagnosed diseases, due to under-coding by physicians. Administrative data may be influenced by misclassification bias. Misclassification of exposures or outcomes can dilute or exaggerate associations, especially if they are different across groups. To overcome this limitation, we used validated algorithms for dementia and covariates. Our dataset did not include people who decided to be vaccinated by private services. However, this phenomenon is quite residual and occurs mostly in younger people being vaccinated within the welfare programs of their companies. Indeed, in people aged 60 or over, Italian authorities provide vaccinations free of charge. Our study considered only one influenza season and did not stratify for other cofounders, including socio-economic factors, educational level, and marital status. We know that social factors are associated with both vaccine acceptance and dementia, so the risk of hospitalization could be under- or overestimated. However, our results are the first data available in the literature from a population-based region-wide cohort in Italy, including all the different socio-demographic groups in the study sample.

## 5. Conclusions

Influenza vaccination plays a crucial role in reducing hospitalizations among older adults. People living with dementia are at higher risk of hospitalization with respiratory diseases. However, in both nursing homes and home-living settings, people living with dementia had a similar vaccination coverage to those without dementia. Public health institutions should consider extending and mentioning dementia as a condition of higher risk and raise awareness of this condition. Vaccine uptake among the elderly with and without dementia is below the minimum goal set by the World Health Organization; thus, tailored policies and interventions are necessary to further protect this vulnerable group and minimize the burden of respiratory diseases in aging populations.

## Figures and Tables

**Table 1:** Number of people vaccinated or not vaccinated among the general population, stratified by different residency settings and dementia status, in Lombardy, over the 2022–2023 influenza season.

	Not Vaccinated	Vaccinated	Total
Overall, n (%)	1,175,454 (48.6)	1,244,825 (51.4)	2,420,279 (100.0)
Residency setting, n (%)			
At home	1,157,738 (49.2)	1,194,285 (50.8)	2,352,023 (100.0)
Nursing home	17,716 (26.0)	50,540 (74.0)	68,256 (100.0)
Dementia, n (%)			
No	1,140,203 (49.0)	1,185,808 (51.0)	2,326,011 (100.0)
Yes	35,251 (37.4)	59,017 (62.6)	94,268 (100.0)

**Table 2:** Number of people vaccinated or not vaccinated among people living with dementia in different residency settings, stratified by sex, in Lombardy, over the 2022–2023 influenza season.

	Not Vaccinated	Vaccinated
At home, n (%)		
Female	16,848 (48.6)	17,841 (51.4)
Male	8442 (43.9)	10,778 (56.1)
Nursing homes, n (%)		
Female	7507 (23.8)	24,071 (76.3)
Male	2454 (27.9)	6327 (72.1)

**Table 3:** Number of people vaccinated or not vaccinated among people without dementia in different residency settings, stratified by sex, in Lombardy, over the 2022–2023 influenza season.

	Not Vaccinated	Vaccinated
At home, n (%)		
Female	627,678 (49.6)	638,075 (50.4)
Male	504,770 (48.9)	527,591 (51.1)
Nursing homes, n (%)		
Female	5484 (27.1)	14,782 (72.9)
Male	2271 (29.8)	5360 (70.2)

## Data Availability

Restrictions apply to the availability of these data. Data were obtained from the Lombardy Region DataWarehouse, only provides data in anonymous and aggregated form. All data handling procedures complied with the European General Data Protection Regulation (GDPR) and local privacy laws to protect the confidentiality and integrity of patient information.

## Supplementary Materials

The following supporting information can be downloaded at: https://www.mdpi.com/article/10.3390/vaccines13050489/s1, Figure S1: Venn diagram with the proportion of people living with dementia according to the hospital discharge records, drug prescriptions, long term facilities and exemptions information flows; Table S1: Algorithm description.
