# Supplementary material for: Influenza Vaccine Uptake and Associated Hospitalization Risk in Older Adults with or Without Dementia: Differences Between at Home-Living and Nursing Home Residents in Lombardy, Italy
Source: Vaccines (Basel). 2025 Apr 30;13(5):489. doi: 10.3390/vaccines13050489 (PMC12115652; doi:10.3390/vaccines13050489)
Supplement: Supplementary file 1 [file vaccines-13-00489-s001.zip › vaccines-3579499-supplementary.pdf]

SUPPLEMENTARY MATERIAL

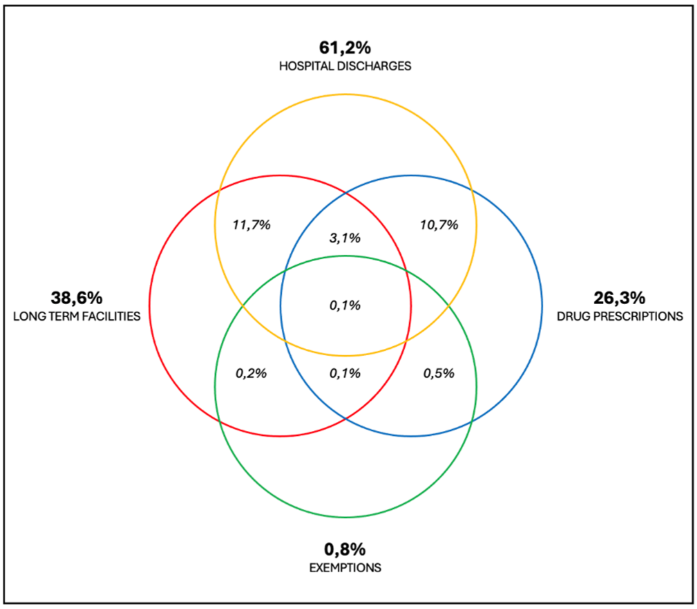

Figure S1. Venn diagram with the proportion of people living with dementia according to the hospital discharge records, drug prescriptions, long term facilities and exemptions information flows.

Table S1. Algorithm description

**Information Flow**	**Description**	**Variables**	**Codes**
SDO	Hospital Discharges Registry	Diagnosis, International Classification of Diseases, 9th Revision, Clinical Modification (ICD-9-CM)	290, 2900, 2901, 29010, 29011, 29012, 29013, 2902, 29020, 29021, 2903, 2904, 29040, 29041, 29042, 29043, 2908, 2909, 2912, 2940, 2941, 29410, 29411, 2942, 29420, 29421, 3310, 3311, 33111, 33119, 3312, 3315, 3317, 3318, 33182, 29282, 0461, 33189, 3319;
FARTER	Drugs Prescriptions	Anatomical Therapeutic Chemical (ATC)	N06DX01, N06DA02, N06DA03, N06DA04;
ESENZIONI	Administrative Exemptions	Exemptions codes	011.290.0, 011.290.1, 011.290.2, 011.290.4, 011.291.1, 011.294.0, 029.331.0
SOSIA	Long-Term Care Facilities admissions	nursing homes code care-setting	6 [Alzheimer's Disease specific])
		Diagnosis, International Classification of Diseases, 9th Revision, Clinical	290, 2900, 2901, 29010, 29011, 29012, 29013, 2902, 29020, 29021, 2903, 2904, 29040, 29041, 29042, 29043, 2908, 2909, 2912, 2940, 2941, 29410, 29411, 2942, 29420, 29421, 3310, 3311, 33111, 33119, 3312, 3315, 3317, 3318, 33182, 29282, 0461, 33189, 3319.

		Modification (ICD-9-CM)	
